# Linguistic Markers of Pain Communication on X (Formerly Twitter) in US States With High and Low Opioid Mortality: Machine Learning and Semantic Network Analysis

**DOI:** 10.2196/67506

**Published:** 2025-05-13

**Authors:** ShinYe Kim, Winson Fu Zun Yang, Zishan Jiwani, Emily Hamm, Shreya Singh

**Affiliations:** 1 Department of Counseling Psychology University of Wisconsin-Madison Madison, WI United States; 2 Department of Psychiatry Massachusetts General Hospital Cambridge, MA United States

**Keywords:** pain communication, opioid mortality, social media, machine learning, semantic network analysis, Linguistic Inquiry and Word Count, LIWC, health communication, digital health, public health surveillance, Twitter

## Abstract

**Background:**

The opioid epidemic in the United States remains a major public health concern, with opioid-related deaths increasing more than 8-fold since 1999. Chronic pain, affecting 1 in 5 US adults, is a key contributor to opioid use and misuse. While previous research has explored clinical and behavioral predictors of opioid risk, less attention has been given to large-scale linguistic patterns in public discussions of pain. Social media platforms such as X (formerly Twitter) offer real-time, population-level insights into how individuals express pain, distress, and coping strategies. Understanding these linguistic markers matters because they can reveal underlying psychological states, perceptions of health care access, and community-level opioid risk factors, offering new opportunities for early detection and targeted public health response.

**Objective:**

This study aimed to examine linguistic markers of pain communication on the social media platform X and assess whether language patterns differ among US states with high and low opioid mortality rates. We also evaluated the predictive power of these linguistic features using machine learning and identified key thematic structures through semantic network analysis.

**Methods:**

We collected 1,438,644 pain-related tweets posted between January and December 2021 using *tweepy* and *snscrape*. Tweets from 2 high-opioid mortality states (Ohio and Florida) and 2 low opioid mortality states (South and North Dakota) were selected, resulting in 31,994 tweets from high-death states (HDS) and 750 tweets from low-death states (LDS). Six machine learning algorithms (random forest, k-nearest neighbor, decision tree, naive Bayes, logistic regression, and support vector machine) were applied to predict state-level opioid mortality risk based on linguistic features derived from Linguistic Inquiry and Word Count. Synthetic Minority Oversampling Technique was used to address class imbalance. Semantic network analysis was conducted to visualize co-occurrence patterns and conceptual clustering.

**Results:**

The random forest model demonstrated the strongest predictive performance, with an accuracy of 94.69%, balanced accuracy of 94.69%, κ of 0.89, and an area under the curve of 0.95 (*P*<.001). Tweets from HDS contained significantly more affective pain words (*t*_31,992_=10.84; *P*<.001; Cohen *d*=0.12), health care access references, and expressions of distress. LDS tweets showed greater use of authenticity markers (*t*_31,992_=−10.04; *P*<.001) and proactive health-seeking language. Semantic network analysis revealed denser discourse in HDS (density=0.28) focused on distress and barriers to care, while LDS discourse emphasized recovery and optimism.

**Conclusions:**

Our findings demonstrated that linguistic markers in publicly shared pain-related discourse show distinct and predictable differences across regions with varying opioid mortality risks. These linguistic patterns reflect underlying psychological, social, and structural factors that contribute to opioid vulnerability. Importantly, they offer a scalable, real-time resource for identifying at-risk communities. Harnessing social media language analytics can strengthen early detection systems, guide geographically targeted public health messaging, and inform policy efforts aimed at reducing opioid-related harm and improving pain management equity.

## Introduction

### Background

Opioids have been the primary treatment for chronic pain [1], although recent years have seen heightened awareness regarding the adverse effects of prolonged opioid use [1] as the nation struggles with the opioid crisis and its dramatic 8-fold surge in opioid-related fatalities since 1999 [2]. While not all opioid use arises from physical pain experiences, individuals living with chronic pain conditions are particularly susceptible to opioid-related complications. Chronic pain impacts approximately 1 in 5 US adults [3] with diminished quality of life, heightened health care expenses, and significantly higher morbidity [4]. Moreover, there are limited therapeutic alternatives to opioids for treatment [5].

One approach to understanding the relationship between chronic pain and opioid-related deaths is to examine how people experiencing the pain articulate it. Historically, chronic pain research has primarily concentrated on its incidence, physical and biological origins or influences, and symptom relief. This emphasis on the origins of physiopathology frequently overlooks the social context in which pain is experienced, although social factors play a pivotal role in the overall pain experience [6].

The social communication model of pain provides a comprehensive perspective on the interplay among biological, psychological, and social elements that mold pain expression [7,8]. This model highlights the multistep pain disclosure process—from pain anticipation and experience to its eventual articulation—shaped by these intertwined factors [9]. Despite the social nature of pain, research often sidelines the significance of social aspects in the pain experience [6]. For instance, those with chronic pain frequently report eroding social connections, increasing feelings of loneliness, and perceiving that others discount their pain [10-12]. As social dynamics decline, communicating about pain becomes increasingly complex. Within this framework, exploring pain disclosure environments becomes vital to identify supportive contexts. Previous studies suggest that social interactions impact pain communication, partner responses, and the overall well-being of individuals enduring chronic pain [13-16]. While a singular, universally shared chronic pain experience is improbable, potential common themes or linguistic patterns in discussions may emerge. These patterns might reflect distinct psychological states and could even serve as indicators of outcomes such as opioid-related deaths. Understanding how language reflects pain experiences in different social and contextual settings may provide insights into broader health disparities and risk factors related to opioid use and mortality.

Because of its widespread use for sharing personal pain experiences and coping strategies, X (formerly Twitter) offers a unique channel for exploring the language and behavior of people living with chronic pain [17]. While all social media platforms have generally seen a significant uptick in health-related discussions, X stands out for its particularly vibrant discourse on chronic pain and related management strategies, including the use of opioids [18]. Researchers have harnessed text-based analyses to collect and study user behaviors, sentiments, and preferences within these online communities, generating invaluable insights. Researchers have leveraged machine learning and natural language processing techniques to analyze opioid-related discussions, detect prescription opioid misuse, and track evolving trends in substance use [19-23]. For instance, Sarker et al [19] successfully built a chronic pain cohort from X data, highlighting the potential of social media for opioid surveillance. Other studies have applied natural language processing methods to detect nonmedical opioid use promotion [20], analyze sentiment in drug-related tweets [21], and identify patterns of polydrug abuse [22]. However, the extent to which linguistic differences in pain-related discourse correspond to opioid mortality risk remains largely unexplored. This study sought to address this gap by applying machine learning classification models and semantic network analysis to assess whether linguistic patterns in pain-related discussions vary among US states with high and low opioid mortality rates.

### Objectives

This paper has 2 primary objectives: (1) to ascertain the predictive power of linguistic features found in pain-related tweets using machine learning and (2) to use semantic network analysis to identify central concepts and themes in chronic pain–related tweets using a text-analysis software program. This paper examines how linguistic patterns in pain-related discussions on social media vary across different US states with high and low opioid mortality. By applying machine learning classification models, we assess whether these differences are predictive of opioid-related outcomes.

## Methods

### Tweet Retrieval and Preprocessing

**Figure 1:**
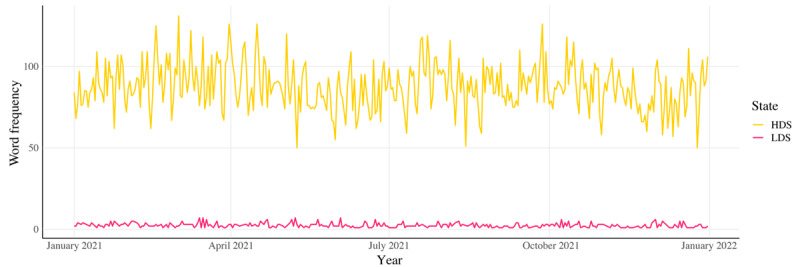
Comparison of daily Twitter activity related to opioid deaths among states with the highest and lowest opioid mortality rates (January 2021-January 2022). The 2 states with the lowest opioid deaths (North and South Dakota) generated 750 tweets in total and 2.83 tweets daily (shown in red). In contrast, the 2 states with the highest opioid deaths (Ohio and Florida) produced 31,994 total tweets and 87.65 daily tweets (shown in yellow). The time series demonstrates consistent patterns where tweets from high-mortality states consistently outnumbered those from low-mortality states throughout the study period, reflecting potential differences in public awareness and discussion of the opioid crisis based on regional impact severity. HDS: high-death states; LDS: low-death states.

The Twitter application programming interface (API) for academics was used to collect tweets from January to December 2021. To analyze linguistic differences in pain-related discussions, we selected tweets from the 2 states with the highest opioid mortality rates (Florida and Ohio) and the 2 with the lowest rates (South and North Dakota) in 2021 [24]. These states were selected strictly based on their opioid mortality rates to ensure that linguistic differences observed in pain-related discussions correspond to opioid mortality risk rather than other demographic or regional factors. We acknowledge that regional news coverage, state policies, and major opioid-related events may influence public discourse and contribute to linguistic differences between states. However, filtering news-driven tweets or distinguishing event-related spikes presents several methodological challenges. First, separating news reports from organic user discussions is complex, as individuals often engage with and react to news in personal tweets. Second, automated event detection requires additional historical datasets and real-time tracking mechanisms that fall outside the scope of traditional linguistic classification approaches. Finally, pain-related and opioid-specific discussions may overlap in nuanced ways that are difficult to disentangle through Linguistic Inquiry and Word Count (LIWC)–based methods alone. While our study does not explicitly filter these factors, future research could explore content-specific classification techniques or event-based filtering to further isolate user-driven narratives from external media influences. While South and North Dakota have smaller populations and lower social media activity compared to Florida and Ohio, their inclusion allows for a meaningful contrast between states at opposite ends of the opioid mortality spectrum. By selecting 2 states per category rather than relying on a single extreme case, we aimed to reduce the influence of state-specific anomalies and provide a more robust analysis of linguistic variations. This approach strengthened the validity of our findings by enhancing comparability across high- and low-mortality states while ensuring that results were not driven by isolated state-level factors. Python (Python Software Foundation) packages *tweepy* [25] and *snscrape* [26] were used to scrape the data. *Tweepy* is a widely used Python library that provides a simple interface to access Twitter’s official API, allowing systematic collection of tweets based on specific search terms. *Snscrape* complements *tweepy* by enabling historical tweet collection beyond the limits of Twitter’s API. Both tools were used in combination to ensure that Twitter data were scraped comprehensively during the selected period. The following chronic pain–related terms were monitored over the year: *chronic pain*, *fibromyalgia*, *headache*, *back pain*, and *migraine*. These words were chosen as they were the top terms used by individuals with chronic pain, based on previous literature [27]. During this period, a maximum of 1000 tweets per day were set for each term, leading to a maximum of 1.825 million possible tweets. In total, we collected a total of 1,438,644 tweets. Due to differences in social media activity between high- and low-mortality states, the dataset was naturally imbalanced, with more tweets originating from high-mortality states (Figure 1). To mitigate potential classification bias, we implemented multiple strategies, such as applying the Synthetic Minority Oversampling Technique (SMOTE) to the training data, evaluating model performance on a held-out test set that preserved the original class distribution, and conducting an additional analysis in which the test set was downsampled to balance the classes. These approaches ensured that model performance was not disproportionately influenced by differences in data availability across states.

### Machine Learning Classification Capacity

Tweets were preprocessed as follows: (1) removing bytes in the text as text were converted to bytes during scraping, (2) transforming all letters to lower case, (3) removing all hyperlinks, (4) removing user mentions, (5) removing hashtag words, (6) removing nonalphanumeric characters, (7) removing additional white spaces, (8) removing emojis, (9) stripping all quotation symbols, and (10) filtering tweets located in Ohio and Florida (high-death states [HDS]) and South and North Dakota (low-death states [LDS]). Due to unbalanced sample sizes in the 2 groups (31,994 from HDS and 750 from LDS), we used SMOTE to upsample the number of tweets in the LDS group to 50% of the tweets in the HDS group, while downsampling the number of tweets in the HDS group to 50% [28].

To examine if linguistic indicators can predict the states with the highest or lowest rate of opioid-related deaths, we used 6 classification methods: random forest, k-nearest neighbor, decision tree, naive Bayes, logistic regression, and support vector machine. Random forest was selected for its ability to combine multiple decision trees to improve accuracy and reduce overfitting and handle complex interactions between high-dimensional linguistic features. k-nearest neighbor is particularly useful for identifying local patterns based on similarity measures between features. Decision tree can capture hierarchical decision rules in features related to sentiments in different states. Naive Bayes is effective in text classification tasks and its ability to handle high-dimensional linguistic data efficiently. Logistic regression provides probabilistic classifications and interpretable feature weights to understand the relative importance of different linguistic markers. Support vector machine is effective in high-dimensional spaces typical of linguistic analysis and its ability to handle nonlinear relationships between features.

We split the data into training (80%) and test (20%) sets, followed by feature selection using recursive feature elimination [29] in the *caret* package [30]. To reduce overfitting, we only selected the top 10 features for training and testing. For all classification algorithms, we conducted a 10-fold cross-validation on the training data to reduce overfitting of the trained data, followed by testing the model on the unseen test datasets.

To compare classification algorithms, we reported the following: (1) accuracy, defined as the number of correct predictions over the total number of predictions, or (TP + TN) / (TP + TN + FP + FN), where TP is true positives, TN is true negatives, FP is false positives, and FN is false negatives; (2) κ, which compares an observed accuracy against the expected accuracy (at random), with higher values representing better classification rate; (3) sensitivity, which is the proportion of true positives that are correctly predicted by the model, and is calculated by TP / (TP + FN); (4) specificity, the proportion of true negatives that are correctly predicted by the model, and is calculated by TN / (TN + FP); (5) precision, which is proportion of positive identification, calculated by TP / (TP + FP); (6) *F*_1_-score, defined as the harmonic mean of the precision and recall (sensitivity), with scores closer to 1 indicating a better model; and (7) area under the curve, which provides an aggregate measure of performance across all possible classification thresholds, with higher values indicating better performance. All machine learning procedures were conducted using the *caret* package in R (R Foundation for Statistical Computing) [30].

As the dataset was naturally imbalanced, with more tweets from high-mortality states, we used multiple strategies to mitigate potential classification bias. To enhance model learning, SMOTE was applied only to the training data, preventing overfitting while allowing the models to better distinguish between high- and low-mortality states based on linguistic features. Importantly, model evaluation was conducted on a held-out test set that preserved the original data distribution, ensuring that classification performance reflected real-world imbalances. In addition, we performed an alternative analysis in which the test set was downsampled to match the number of samples in the LDS group, allowing us to compare classification outcomes across different sampling strategies. These complementary evaluation methods provide a robust assessment of classification performance and reduce potential biases introduced by synthetic oversampling. This approach allowed us to report three sets of performance metrics: (1) performance in the SMOTE dataset, reflecting an ideal performance; (2) performance on the original test set distribution, reflecting real-world performance where class imbalance exists; and (3) performance on the balanced test subset when controlling for class imbalance effects. For this set of analyses, we also reported balanced accuracy, a metric used to evaluate the performance of a classification model, especially when dealing with imbalanced datasets. Balanced accuracy is calculated as the average of the sensitivity and specificity.

### Semantic Network Analysis

The same tweets used in the machine learning analyses were used for semantic network analysis, with slightly different preprocessing steps: (1) ensuring text were in lower case, (2) removing punctuation, (3) removing alphanumeric characters, (4) removing numerics, (5) removing non-English words, (6) removing hyperlinks, (7) removing hashtags, (8) removing emojis, (9) removing remaining bytes characters, (10) removing additional white spaces, and (11) reducing words to their stems (eg, reducing “trouble,” “troubles,” and “troubling” to “trouble*”). This process resulted in 5661 usable word stems across 1500 tweets.

### LIWC Program

We used LIWC (version 2015) [31], a well-established psycholinguistic lexicon software program, to categorize tweets into groups of predefined categories using the program’s built-in dictionaries [31]. LIWC reads the text and compares each word against a user-defined dictionary to categorize associated words into the relevant categories. Then, it calculates the percentage of total words that match each of the dictionary categories. In this study, we used the entire LIWC dictionary and user-defined pain dictionary [9], creating 82 linguistic features, subfeatures, and descriptors. The pain dictionary can identify sensory and affective pain words and has shown validity in the original study [9]. The categories for pain-related words included: (1) Pain: Total, (2) Pain: Sensory, (3) Pain: Affect, (4) Pain: Sensory-affective, and (5) Pain: Medical. The LIWC 2015 manual provides more details about each LIWC category [31].

### Construction of Semantic Network

Semantic networks are graph-based analyses of relationships of written text, in which data are structured as a network of words that co-occur or are related to one another in the text [32]. Within the network, nodes are words that represent specific concepts in the text, while edges represent relationships between concepts or units of meaning. In the context of this study, the semantic networks formed are knowledge and concepts centered around chronic pain. In an exemplar tweet, “Pain is giving me a bad day,” pain, bad, and day are nodes and pain-bad, pain-day, and bad-day are edges, as they are concepts that appear together in the tweet. Meaningful ideas or themes centered around chronic pain can also be extracted from semantic networks based on how concepts cluster together.

We created a sparse matrix of word connections by first constructing a text matrix of words, followed by filtering word connections with at least 20 words. Words that do not appear together in the same text at least 20 times were coded as 0. Edges were created for words that occurred within 5 words of one another within each tweet as studies suggest that typical individuals only process 3 to 5 meaningful bits of information at a time [33].

### Network Metrics

We used the Louvain community detection method to reveal clusters of words within a network, with each cluster indicated by a different color [34]. The Louvain method is a popular community detection method in network science that is used to identify communities within a graph. This method optimizes modularity, a measure that quantifies the strength of separation of a network into communities. The Louvain community detection method is efficient for large networks such as social media data and is able to detect the optimal number of communities. In addition, several network metrics were calculated, including network density, node degree (DN), degree centrality (CD), betweenness centrality (CB), closeness centrality (CC), and eigenvector centrality (CE). These metrics describe how central and connected words are within the network. Network density describes the interconnectedness of nodes, which is defined as the number of edges divided by the number of possible edges in the network. Higher values indicate that more word concepts are intertwined and that the discussion around the topic is more complex.

DN is the number of links connecting each word. CD describes how well a concept is connected to other concepts in the network. It is measured by the number of connections normalized by the total number of network connections. CB describes the likelihood that one concept connects 2 other concepts in the network. Higher CB indicates that concept A will pass through concept B while going to concept C. In other words, it detects information flow in the semantic network. CC measures closeness of concepts by calculating the inverse of the sum of the shortest paths between a concept to all other concepts in the network. In other words, concepts with higher CC are interpreted to be more central than concepts as they have shorter network distance on average compared to other concepts. Finally, CE is a more complex measure for assessing NDs. CE acknowledges that not all concepts and their connections have equal weight and, therefore, assigns relative scores to all concepts in the network based on the number and quality of its connections to other concepts. In general, connections to high-scoring concepts contribute more to the score of the concept than equal connections to low-scoring concepts. CE indicates a concept’s relative influence or how central it is in the network. The semantic networks were constructed using the *tm* [35,36] and *igraph* [37] packages in R. The network metrics and network plots were conducted using *Gephi*, an open-source software for network analysis [38].

### Ethical Considerations

This study involved the collection and analysis of publicly available data from X (formerly Twitter) shared by users on an open platform. All collected data were fully anonymized by removing usernames, account handles, profile information, and any other personally identifiable information. Analyses were conducted solely on tweet content, with no examination of user identities. This study was not considered human subjects research and therefore did not require institutional review board approval, as it involved the analysis of publicly available data.

## Results

### Machine Learning Classification

The performance metrics of 6 machine learning models were evaluated under 3 distinct sampling scenarios: the original imbalanced dataset, SMOTE application to the entire dataset, and SMOTE application to training data with downsampling of test data (Table 1**)**.

**Table 1:** Metrics for different classification methods.

Use case scenario and model		Accuracy (%)	*P* value			Balanced accuracy (%)	*P* value			κ		Sensitivity (%)		Specificity (%)		Precision (%)		*F*_1_-score		AUC^a^
**SMOTE^b^ applied on entire data to present a balanced dataset**																				
	Random forest	94.69		<.001	94.69			<.001	0.89		94.69		94.69		94.69		0.95		0.95	
	k-nearest neighbor	81.81		<.001	81.81			<.001	0.64		66.02		97.59		96.48		0.78		0.85	
	Decision tree	69.26		<.001	69.26			<.001	0.39		61.61		76.9		72.73		0.67		0.7	
	Naive Bayes	60.63		<.001	60.63			<.001	0.21		44.58		76.68		65.65		0.53		0.62	
	Logistic regression	55.67		<.001	55.67			<.001	0.11		47.92		63.43		56.71		0.52		0.56	
	Support vector machine	54.5		<.001	54.5			<.001	0.09		51.86		57.14		54.75		0.53		0.55	
**Original dataset without SMOTE on training data**																				
	Random forest	97.77		.39	51.98			.39	0.07		99		4		97.8		0.99		0.52	
	k-nearest neighbor	97.71		.52	0.5			.52	0		100		0		97.71		0.99		0.5	
	Decision tree	97.71		.59	0.5			.59	0		100		0		97.71		0.99		0.5	
	Naive Bayes	97.68		.52	49.98			.52	−0.006		99		0		97.71		0.99		0.5	
	Logistic regression	97.71		.52	50			.52	0		100		0		97.71		0.99		0.5	
	Support vector machine	97.71		.52	50			.52	0		100		0		97.71		0.99		0.5	
**SMOTE applied on training data with downsampling on test data to present a balanced dataset**																				
	Random forest	55		.046	55			.046	0.1		95.33		14.67		52.77		0.68		0.55	
	k-nearest neighbor	53.67		.66	53.67			.66	0.07		39.33		68		51.4		0.46		0.54	
	Decision tree	52.33		.07	52.33			.07	0.05		52.67		52		52.32		0.52		0.52	
	Naive Bayes	54.33		.09	54.33			.09	0.09		67.33		41.33		53.44		0.6		0.54	
	Logistic regression	49		.23	49			.23	−0.02		56.00		42		49.12		0.52		0.49	
	Support vector machine	54		.11	54			.11	0.08		48.67		59.33		54.48		0.51		0.54	

^a^AUC: area under the curve.

^b^SMOTE: Synthetic Minority Oversampling Technique.

Table 1 outlines the performance metrics of 6 machine learning classification models using 10-fold cross-validation to predict states with HDS or LDS based on pain-related tweets in these states across 3 different sampling strategies. It compares algorithm performance under (1) SMOTE-balanced entire dataset, (2) original imbalanced dataset (97.7% negative class), and (3) SMOTE-balanced training data with downsampled test data. Random forest consistently outperformed other algorithms, achieving 94.69% balanced accuracy with SMOTE applied to the entire dataset.

In the original imbalanced dataset scenario, all models demonstrated exceptionally high accuracy (97.68%-97.77%) but exhibited poor discriminative ability. The balanced accuracy ranged from 0.50% to 51.98%, with near-zero or negative κ values (−0.0006 to 0.07). While sensitivity was consistently high (99%-100%), specificity was notably poor (0%-4%), indicating that models predominantly predicted HDS. The random forest classifier marginally outperformed other models with 4% specificity, although this still indicates insufficient practical utility.

When SMOTE was applied to the training data with downsampling of the test data, overall accuracy metrics decreased substantially but showed improved balance between LDS and HDS. The random forest classifier achieved the highest accuracy at 55.00% (*P*=.046), with 95.33% sensitivity and 14.67% specificity. Other models demonstrated comparable performance, with accuracies ranging from 49% to 54.33%. Notably, naive Bayes showed relatively balanced performance with 67.33% sensitivity and 41.33% specificity, resulting in an *F*_1_-score of 59.59%.

**Figure 2:**
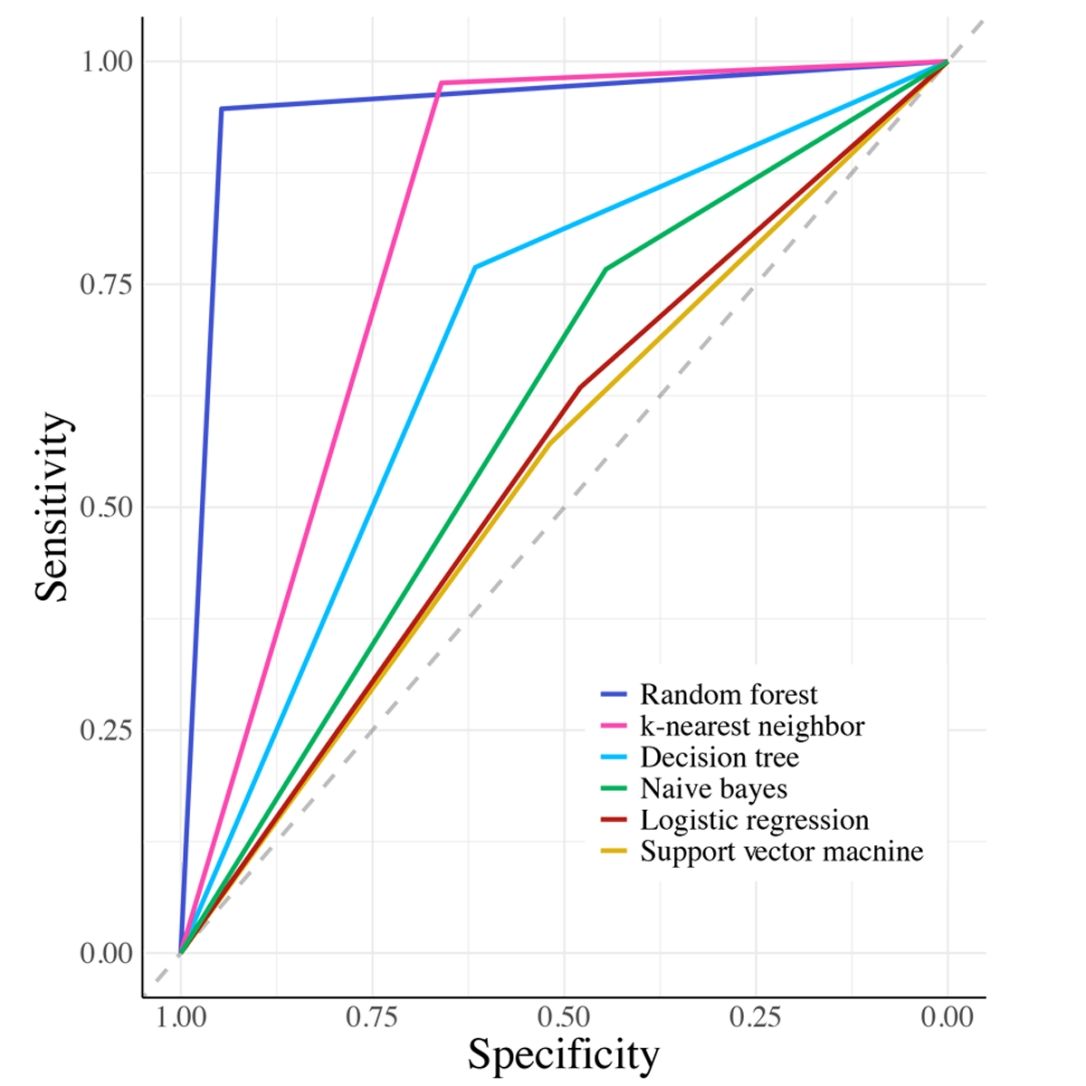
Receiver operating characteristic curves comparing the performance of 6 machine learning classification algorithms. Random forest demonstrated superior performance (area under the curve [AUC]=0.95), followed by k-nearest neighbor (AUC=0.85), decision tree (AUC=0.70), naive Bayes (AUC=0.62), logistic regression (AUC=0.56), and support vector machine (AUC=0.55). The diagonal dashed line represents random classification (AUC=0.50).

The application of SMOTE to the entire dataset yielded the most robust performance across all models. The random forest classifier demonstrated superior performance with 94.69% accuracy (*P*<.001), 0.89 κ coefficient, and 94.69% for both balanced sensitivity and specificity. The k-nearest neighbor algorithm ranked second in performance, achieving 81.81% accuracy (*P*<.001) with notably high specificity (97.59%) but lower sensitivity (66.02%). Decision tree, naive Bayes, logistic regression, and support vector machine models showed progressively decreasing performance, with accuracies ranging from 69.26% to 54.5% (*P*<.001). The area under the curve for this model is shown in Figure 2.

Across all sampling strategies, the random forest classifier consistently outperformed other models, particularly when SMOTE was applied to the entire dataset. However, it is important to note that while this sampling strategy produced the most favorable metrics, the application of SMOTE to the entire dataset, including test data, may result in optimistically biased performance estimates due to the introduction of synthetic samples in the evaluation set.

The feature importance for random forest classification were reported as follows, with higher values indicating higher importance: preposition (100), authentic (88.83), affect (47.52), relativity (43.36), analytic (41.31), verb (36.63), function (28.52), pain: total (23.98), health (9.24), and biological (0) words. Notably, tweets from high-opioid mortality states showed greater use of affective pain words, references to health care access, and expressions of distress (eg, “suffer,” “need,” “hurt”). In contrast, tweets from low opioid mortality states contained more words reflecting well-being, social engagement, and proactive treatment-seeking (eg, “better,” “health,” “doctor”).

To further support our results, we conducted several 2-tailed *t* tests to examine group differences in the word frequencies in each category. The *t* tests revealed that the HDS group used more pain: total (t_31,992_=17.42; *P*<.001; Cohen d=0.19; 95% CI 1.22-1.53), health (t_31,992_=16.03; *P*<.001; Cohen d=0.18; 95% CI 1.20-1.54), biological processes (t_31,992_=14.31; *P*<.001; Cohen d=0.16; 95% CI 1.15-1.52), analytic (t_31,992_=5.93; *P*<.001; Cohen d=0.07; 95% CI 1.37-2.74) and affective (t_31,992_=10.84; *P*<.001; Cohen d=0.12; 95% CI 0.67-0.96) words than the LDS group. In contrast, the LDS group used more authentic (t_31,992_=−10.04; *P*<.001; Cohen d=0.11; 95% CI −4.90 to 3.30), relativity (t_31,992_=−3.71; *P*=.002; Cohen d=0.04; 95% CI −0.52 to 0.16), function (t_31,992_=15.58; *P*<.001; Cohen d=0.17; 95% CI −2.53 to 1.97), prepositions (t_31,992_=8.33; *P*<001; Cohen d=0.09; 95% CI −0.69 to 0.43), and verb-related (t_31,992_=7.01; *P*<.001; Cohen d=−0.08; 95% CI −0.72 to 0.41) words than those in the HDS group.

### Semantic Network Analysis

**Figure 3:**
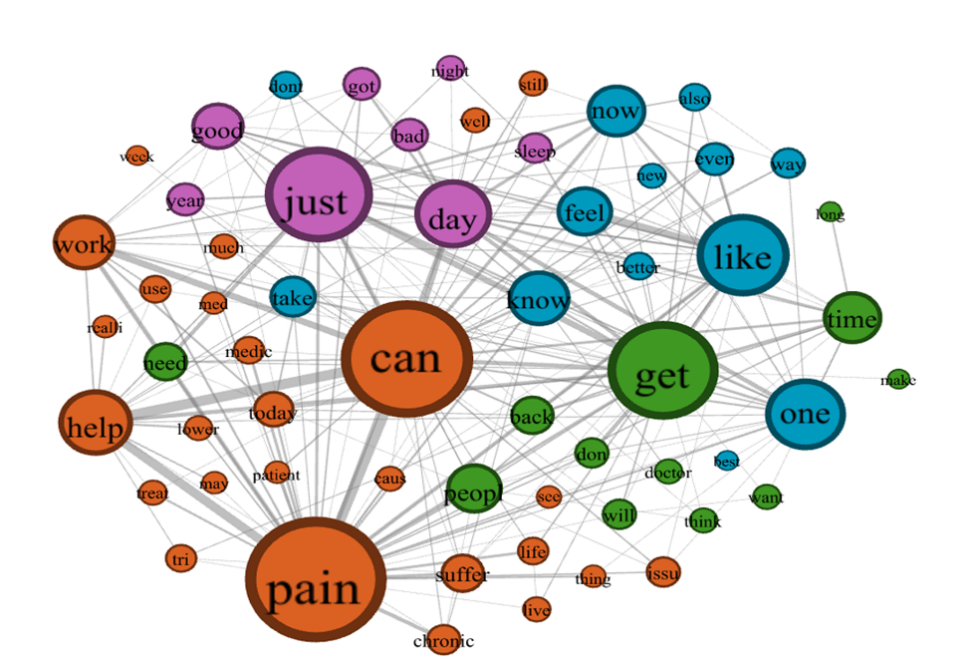
Semantic network visualization of word co-occurrences in social media discussions about chronic pain in states with high-opioid mortality rates (high-death states). Node sizes represent degree centrality, with larger nodes indicating words more frequently connected to other concepts. Edge thickness indicates co-occurrence frequency between connected words, where thicker lines represent higher frequency of the 2 concepts occurring in the same tweet. The network reveals 4 distinct community clusters: medical or suffering concepts (orange), health care engagement (green), coping mechanisms (blue), and temporal experiences (purple). The most prominent nodes, “pain,” “can,” “get,” “just,” “like,” and “day,” highlight the central themes in chronic pain discussions: the experience of pain itself, coping capabilities, recovery efforts, and temporal aspects of the condition.

**Figure 4:**
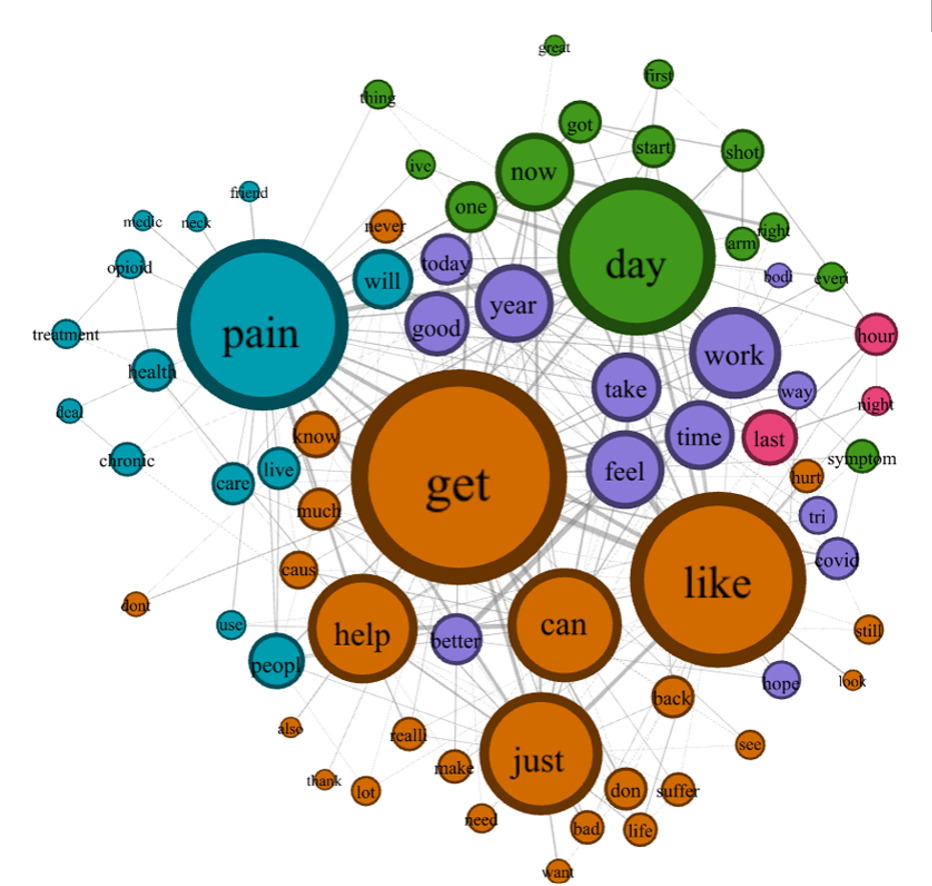
Semantic network visualization of word co-occurrences in chronic pain discussions in social media from regions with low opioid mortality rates (low-death states). Node sizes represent degree centrality, with larger nodes indicating words more frequently connected to other concepts. Edge thickness indicates co-occurrence frequency between connected words, where thicker lines represent higher frequency of the 2 concepts occurring in the same tweet. The network reveals 5 distinct community clusters: health care resources (blue), pain experiences (orange), physical symptoms (green), experiential descriptors (purple), and temporal aspects (pink). The most prominent nodes, “pain,” “get,” “day,” “like,” “just,” and “can,” highlight the focus on pain management and daily coping mechanisms in low opioid mortality regions. The conversational patterns suggest emphasis on functional management rather than medication-centric approaches, with stronger connections between pain experiences and daily activities.

The semantic networks for the HDS and LDS groups are shown in Figures 3 and 4, respectively. Properties of the 2 semantic networks are summarized in Table 2. For the HDS group, the number of concepts (DN=59) and connections (480 edges) were fewer than the LDS group (DN=59 and 548 edges). The HDS semantic network also had fewer community clusters (4) than the LDS semantic network (5 clusters). However, the HDS network was denser (network density=0.28) than the LDS semantic network (network density=0.22), suggesting that discussions about chronic pain in high-opioid mortality states were more concentrated around pain, distress, and coping struggles, while discussions in low opioid mortality states were more dispersed, incorporating broader themes such as health care access and recovery. Furthermore, the HDS semantic network had higher CD (0.14), CB (0.018), CC (0.50), and CE (0.31) than the LDS semantic network (C_D_=0.11; C_B_=0.016; C_C_=0.48; C_E_=0.26).

**Table 2:** A summary of network metrics for text networks on chronic pain.

Chronic pain sentiment	Highest	Lowest
Total number of tweets	31,994	750
Number of tweets used in analysis	750	750
Total number of nodes in network	59	70
Total number of edges in network	480	548
Network density	0.28	0.22
Node degree, mean (SD)	8.14 (8.78)	7.83 (8.82)
Weighted node degree, mean (SD)	55.93 (69.13)	52.74 (66.93)
Degree centrality, mean (SD)	0.14 (0.15)	0.11 (0.13)
Betweenness centrality, mean (SD)	0.018 (0.05)	0.016 (0.05)
Closeness centrality, mean (SD)	0.50 (0.08)	0.48 (0.07)
Eigenvector centrality, mean (SD)	0.31 (0.24)	0.26 (0.21)
Number of clusters	4	5

The “Highest” column represents data from Ohio and Florida (31,994 total tweets, random sample of 750 analyzed; HDS), while the “Lowest” column represents North and South Dakota (750 total tweets, all analyzed; LDS). Despite the significant difference in overall tweet volume, the semantic networks show interesting structural variations: LDS demonstrated more complex lexical diversity (70 vs 59 nodes) and more interconnections (548 vs 480 edges), although with lower network density (0.22 vs 0.28). HDS exhibited slightly stronger centrality measures across all metrics, suggesting more concentrated discourse patterns around key terms. Communities revealed 4 thematic clusters in HDS versus 5 in LDS, indicating potentially more diversified discussion topics in regions less affected by opioid deaths.

The concepts in each community cluster are shown in Table 3.

**Table 3:** Thematic word clusters identified in chronic pain Twitter discussion from states with highest versus lowest opioid death states.

Thematic clusters identified in different states			Word clusters
**Highest opioid death states**			
	Medical, pain, or distress-related concepts	can, caus, chronic, help, *issu*^a^, life, live, *lower*, *may, med*, medic, much, pain, *patient,* realli, see, still, suffer, thing, today, *treat,* tri, use, *week, well,* work	
	Coping mechanisms	also, *best,* better, dont, *even,* feel, know, like, *new,* now, one, take, way	
	Temporal experiences	bad, day, good, got, just, night, *sleep,* year	
	Health care engagement	back, *doctor,* don, get, *long,* make, need, peopl, *think,* time, want, will	
**Lowest opioid death states**			
	Health care resources	*care,* chronic, *deal, friend, health,* live, medic, *neck, opioid,* pain, peopl, *treatment,* use, will	
	Pain experiences	also, back, bad, can, caus, don, dont, get, help, *hurt,* just, know, life, like, *look, lot,* make, much, need, *never,* realli, see, still, suffer, *thank,* want	
	Temporal aspects	*hour, last,* night	
	Physical symptoms	*arm,* day, *everi, first,* got, *great, ive,* now, one, *right, shot, start, symptom,* thing	
	Experiential descriptors	better, *bodi, covid,* feel good, *hope,* take, time, today, tri, way, work, year	

^a^Italicized terms represent unique concepts not appearing in the corresponding network, highlighting regional variations in pain discourse.

Table 3 displays community clusters detected using the Louvain algorithm, revealing distinctive conceptual groupings. HDS (Ohio and Florida) exhibited 4 main thematic clusters: (1) medical, pain, or distress-related concepts, (2) coping mechanisms, (3) temporal experiences, and (4) health care engagement. LDS (North and South Dakota) demonstrated 5 clusters: (1) health care resources, (2) pain experiences, (3) temporal aspects, (4) physical symptoms, and (5) experiential descriptors. Italicized terms represent unique concepts not appearing in the corresponding network, highlighting regional variations in pain discourse. Notable distinctions include medical terminology prominence in high-mortality states (“patient,” “med,” “treat”) versus more resource-focused language in low-mortality regions (“care,” “friend,” “treatment”).

Tables 4 and 5 summarize the top 20 words for several centrality measures in the HDS and LDS semantic networks, respectively. The top 10 concepts for the HDS semantic network included *pain*, *can*, *get*, *just*, *like*, *one*, *day*, *help* (common among all centrality measures), *work*, *today* (C_D_), *time*, *know* (C_B_ and C_C_), *work*, and *feel* (C_E_). The most central 10 concepts of the LDS semantic network were *get*, *day*, *pain*, *like*, *just*, *help*, *can*, *work*, *feel* (common among all centrality measures), *now* (C_D_ and C_B_), and *year* (C_C_ and C_E_). Unique concepts among the top 10 concepts found in the HDS semantic network were *one*, *today*, *time*, and *know*; and *now* and *year* for the LDS semantic network. Expanding to the top 20 concepts, unique concepts found in the HDS semantic network were *back*, *need*, *suffer*, *sleep*, and *much*. In contrast, unique concepts found in the LDS semantic network were *better*, *year*, *last*, *health*, and *don*.

**Table 4:** Top 20 most central nodes and centrality measures for the high-death states semantic network.

	Degree centrality				Betweenness centrality				Closeness centrality			Eigenvector centrality	
	Concept		Centrality	Concept		Centrality		Concept		Centrality	Concept		Centrality
1	Pain	40		Pain			0.25	Pain		0.76	Pain		1.00
2	Can	37		Can			0.21	Can		0.73	Can		0.97
3	Get	30		Get			0.14	Get		0.67	Just		0.86
4	Just	29		Just			0.11	Just		0.67	Get		0.86
5	Like	24		One			0.075	Like		0.63	Like		0.78
6	One	20		Like			0.052	One		0.60	One		0.66
7	Day	19		Time			0.050	Day		0.60	Day		0.64
8	Help	18		Day			0.047	Help		0.59	Work		0.55
9	Work	14		Help			0.043	Time		0.56	Help		0.55
10	Know	14		Know			0.019	Know		0.56	Feel		0.54
11	Now	13		Now			0.017	Now		0.56	Know		0.51
12	Time	13		Work			0.012	Work		0.56	Now		0.49
13	Feel	12		People			0.0097	Feel		0.56	Time		0.49
14	People	12		*Suffer* ^a^			0.0042	People		0.55	People		0.47
15	Good	11		*Back*			0.0040	Take		0.54	Take		0.46
16	Take	9		Feel			0.0037	Good		0.53	Good		0.44
17	*Back*	8		Will			0.0036	*Suffer*		0.53	*Need*		0.37
18	*Need*	8		Good			0.0035	*Need*		0.52	*Suffer*		0.36
19	*Suffer*	8		*Sleep*			0.0029	*Back*		0.52	Today		0.35
20	Today	40		*Much*			0.0018	Today		0.52	*Back*		0.34

^a^Italicized words are concepts that are unique to the high-death states' semantic network.

**Table 5:** Top 20 central nodes and centrality measures for the low-death states semantic network.

	Degree centrality				Betweenness centrality				Closeness centrality			Eigenvector centrality	
	Concept		Centrality	Concept		Centrality		Concept		Centrality	Concept		Centrality
1	Get	45		Get			0.26	Get		0.74	Get		1
2	Like	36		Pain			0.23	Like		0.68	Day		0.82
3	Pain	35		Like			0.18	Pain		0.67	Pain		0.81
4	Day	32		Day			0.11	Day		0.65	Like		0.80
5	Just	24		Just			0.077	Just		0.61	Just		0.65
6	Can	22		Help			0.066	Help		0.58	Help		0.58
7	Help	21		Can			0.047	Can		0.58	Can		0.57
8	Work	17		Now			0.039	Work		0.56	Work		0.52
9	Feel	14		Work			0.028	Feel		0.55	Feel		0.52
10	*Year* ^a^	14		Feel			0.013	*Year*		0.55	*Year*		0.51
11	Now	14		Good			0.0095	Now		0.55	Now		0.46
12	Take	12		*Last*			0.0088	Take		0.53	Time		0.42
13	Time	12		Take			0.0088	*Last*		0.52	Take		0.41
14	Good	11		*Year*			0.0083	People		0.52	Good		0.37
15	Will	10		Time			0.0075	Time		0.52	Will		0.37
16	*Last*	9		*Health*			0.0069	Know		0.52	*Last*		0.35
17	People	9		*Don*			0.0067	Will		0.52	People		0.34
18	*Better*	8		Know			0.0047	One		0.51	One		0.33
19	Today	8		People			0.0039	Today		0.51	Today		0.33
20	One	8		*Better*			0.0038	*Better*		0.51	Know		0.32

^a^Italicized terms highlight concepts uniquely prominent in low-death states' semantic network.

Table 4 presents CD (number of connections), CB (bridging role between concepts), CC (proximity to all other concepts), and CE (connection to other high-influence concepts) for the top 20 concepts. Italicized words are concepts that are unique to the highest semantic network, that is, concepts not found in the top 20 centrality measures in the lowest semantic network. “Pain” consistently ranked highest across all centrality measures, followed by action-oriented terms “can” and “get,” indicating discourse focused on coping capabilities. Italicized terms highlight concepts uniquely prominent in HDS compared to LDS, with “suffer,” “back,” “sleep,” and “much” appearing as distinctive bridge concepts (CB). This network structure suggests that pain discussions in HDS emphasize pain-related hardship experiences and physical symptoms, with stronger interconnections between pain-related terminology and emotional expressions.

Table 5 presents CD, CB, CC, and CE for the top 20 concepts. Unlike in HDS, “get” consistently ranked highest across all centrality measures, followed by “like” and “day,” indicating discourse focused on daily management and coping strategies rather than the pain experience itself. Italicized terms highlight concepts uniquely prominent in LDS, with “year,” “last,” “health,” “don,” and “better” appearing as distinctive concepts not found among the top centrality measures in HDS. This network structure suggests that pain discussions in LDS emphasize temporal aspects, health management, and improvement possibilities, with stronger interconnections between coping terminology and forward-looking expressions.

## Discussion

### Principal Findings

Given the significant public health and economic toll of the opioid epidemic [39], the ability to discern variations between states with high and low opioid death rates could prove invaluable to public health officials and researchers. Previous studies have pinpointed X as a platform where psychological signals can be gleaned from digital footprints via language analysis [40]. Evidence suggests that many individuals use social media to share their pain experiences with their social circles [9]. We pursued 2 objectives: first, to determine the most effective algorithm for predicting variations in chronic pain–related tweets from states with the highest and lowest opioid death rates and, second, to identify linguistic disparities between HDS and LDS states through semantic network analysis.

Our examination of pain narratives on X appears to corroborate the multistep pain disclosure process proposed by Craig [6] and Hadjistavropoulos et al [8]. Tweets from high-opioid mortality states exhibited greater use of words associated with pain, distress, and frustration, reflecting heightened emotional intensity in pain-related discussions. In contrast, tweets from low opioid mortality states more frequently referenced health, medical care, and recovery, suggesting a greater focus on proactive coping and support-seeking. These findings highlight the role of social and contextual factors in shaping pain communication, with potential implications for opioid prevention and intervention efforts. It is evident that chronic pain discussions are not just reflective of physiological distress but also deeply intertwined with the social experiences of those affected by chronic pain. Linguistic patterns extracted from our Twitter data hint at potential universal themes in how pain is communicated. Such themes could correspond to specific psychological and social states, possibly providing insights into outcomes such as opioid mortality. In this research, we used 6 machine learning algorithms to classify tweets from either HDS or LDS based on sentiments related to chronic pain. We tested algorithms, including random forest, k-nearest neighbor, decision tree, naive Bayes, logistic regression, and support vector machine. The results highlighted that the random forest algorithm was particularly adept at predicting states with the highest or lowest opioid-related deaths based on linguistic tweet patterns.

Among the tested algorithms, random forest achieved an accuracy of 94.69%, while the support vector machine lagged at 54.5%. While tree-based methods such as decision trees and random forests are generally preferred for multiclass classification (>2 groups), our results indicated a deviation from this trend. This might be due to the design of the random forest algorithm, which amalgamates several decision trees, each based on different randomly selected subsets of variables, thereby introducing a degree of randomness [41]. This makes it sturdy against noise and outliers. In contrast, the support vector machine, as suggested by Domingos [42], might not capture the intricate patterns in the training data, especially when dealing with complex data such as social media content. This echoes findings from other research, as random forest has been favored in sentiment classification due to its balance between efficiency, comparability, and accuracy [43]. However, it is essential to underscore that data attributes, such as its size, type, and other defining characteristics, can influence the choice of the most fitting machine learning algorithm.

This study highlighted distinct differences in language use regarding chronic pain–related tweets between US states with the highest rates of opioid deaths (Florida and Ohio) compared to those with the lowest rates (South and North Dakota). While pain encompasses a broad spectrum of sensations [44], the specific language used to describe pain has been somewhat overlooked in the literature [9]. Our findings revealed that in states with higher death rates, individuals used more words linked to pain, biological processes, and affect than in states with lower death rates. A heightened use of affective words when discussing pain has been previously associated with a stronger inclination to seek social support [45]. In this study, Twitter users in HDS appeared to demonstrate more analytical thinking, characterized by language that is logical, formal, or hierarchical. Such language tends to be impersonal, rigid, and distant [46]. On the other hand, Twitter users in LDS displayed more authentic language use, which is generally more personal, humble, and honest [31]. This suggests that users in HDS are more socially cautious, perhaps being more selective in their word choice and providing less disclosure of their genuine thoughts and emotions. Given the previously proposed idea that language significantly influences the experience of pain [47], pinpointing specific language patterns tied to greater risk could be invaluable for public health officials.

The semantic network in HDS was more cohesive and interconnected versus the LDS network. This indicates that sentiments on chronic pain are more unified for individuals residing in states with higher opioid death rates. Such increased cohesion and interconnectedness suggest that individuals in these states might share more common experiences and perceptions related to chronic pain. This consolidated perspective could arise from factors such as heightened exposure to opioid use and abuse, better access to pain management resources, or various sociocultural influences.

The centrality measures shed light on key concepts that are prominently featured in discussions of chronic pain for each group. For instance, the high centrality of the term “pain” in both groups underscores its universal significance. However, words such as “get” and “just” had greater centrality in the LDS semantic network, possibly indicating a tendency toward seeking immediate solutions instead of addressing the underlying causes of chronic pain.

Distinct concepts in each group can offer insights into the specific concerns and priorities of individuals from these states. For instance, the term “sleep” is notably present only in the HDS semantic network, suggesting that individuals from those states might be particularly concerned about how chronic pain affects sleep. Conversely, the term “health” is among the top 20 concepts for states with low rates of opioid deaths, hinting at a broader concern for overall well-being rather than merely pain management. The HDS semantic network featured clusters related to coping mechanisms, emotional experiences, and the influence of chronic pain on daily life. In contrast, the LDS network focused on clusters pertaining to pain management, health care accessibility, and support systems. This study not only aligns with previous findings that suggest pain-related discussions occur on social media [9] but also expands on earlier research by illustrating potential variances in language use depending on context.

The analysis of linguistic patterns in pain-related tweets uncovers significant distinctions between regions with high and low opioid death rates, providing a valuable perspective for health care professionals. A central insight for clinicians is the marked emphasis on the physiological aspects of pain, as evidenced by the HDS group’s increased use of words related to pain, health, and biological processes. Recognizing these linguistic subtleties can empower health care providers to fine-tune their patient interactions and interventions. By identifying the key linguistic markers of individuals at heightened risk for opioid-related complications, clinicians can better strategize early interventions. Moreover, this study highlights the necessity for a comprehensive approach to pain management. Beyond merely targeting physiological relief, there is a pressing need to incorporate psychological and social facets into pain management plans. Doing so can diversify treatment approaches and diminish the sole dependence on opioids.

This study, leveraging the vast expanse of social media, particularly Twitter, introduces a novel paradigm in discerning patterns related to opioid deaths, providing a fresh avenue for public health researchers. The efficacy of machine learning models, notably the random forest in differentiating between high and low opioid death regions, underscores the untapped potential of such methodologies in health research. The linguistic distinctions between the HDS and LDS groups, especially the varied centrality of certain words, pose intriguing avenues for future exploration. It is imperative for subsequent studies to probe these linguistic nuances more deeply, examining their direct and indirect ties with opioid consumption behaviors. Furthermore, expanding such analyses across diverse platforms, conditions, or demographics could yield a richer, holistic perspective, deepening our grasp of the complex interplay between language, pain, and opioid use.

The insights this study offers concerning the nexus between linguistic markers and opioid-related fatalities can empower policy makers. By vigilantly monitoring and dissecting public discourse on platforms such as Twitter, governmental bodies and health organizations stand a chance to identify regions or communities on the brink of an opioid misuse or overdose outbreak. The subtle variations in words and themes prevalent in areas with a high rate of opioid fatalities can critically inform public health initiatives. For these campaigns to achieve maximal resonance, they must align both linguistically and contextually with their target audience. Furthermore, these revelations pave the way for sculpting public health interventions that are not merely directive but also deeply empathetic, acknowledging the intricate mesh of psychological and social determinants influencing opioid misuse. Such insights herald a more enlightened, proactive, and comprehensive strategy to address the opioid epidemic.

Our findings align with the principles of narrative medicine, which emphasize the importance of patient storytelling in understanding health experiences and informing care [48,49]. The linguistic distinctions observed in high and low opioid mortality states suggest that social media serves as a platform where individuals construct and share their lived experiences of pain. This aligns with previous work suggesting that digital storytelling plays a role in shaping patient identities and public health discourse [17]. Future research could explore how these web-based narratives influence self-perception, social support-seeking, and engagement with health care systems, further bridging the gap between digital linguistics and narrative medicine.

### Limitations, Implications, and Future Direction

This study possesses several limitations that need to be acknowledged when interpreting its results. Using Twitter data as a surrogate for chronic pain experiences might not faithfully represent the broader population. Twitter’s user demographic leans toward younger, tech-savvy individuals, which may not mirror the segment most impacted by chronic pain. Furthermore, our emphasis on English-language tweets potentially overlooks non-English-speaking communities that grapple with chronic pain.

A notable disparity exists in the volume of tweets from HDS (31,994) compared to those from LDS (750). Although the SMOTE technique was used to balance the dataset by upsampling the LDS and downsampling the HDS, this disparity might have posed challenges for the machine learning algorithms. The contrast between sampling scenarios, where imbalanced data produced misleadingly high accuracy and balanced data provided more modest yet reliable results, underscores the complexity of social media–based public health research. While random forest classification performed well with complete SMOTE application, the most methodologically sound approach—using SMOTE only in training—suggests that linguistic markers can aid in distinguishing between high and low opioid mortality states but should be interpreted within the broader context of opioid sentiment analysis.

Instead of using natural language processing to classify text sentiments directly, we used LIWC to categorize sentiments, which can lose the nuanced high-dimensionality of tweets. Text sentiments often harbor subtleties that LIWC’s manual coding might miss. For instance, phrases such as “damn good steak” could be misinterpreted by LIWC due to the juxtaposition of “damn” (negative) with “good” (positive) [43]. Raw text data processed through machine learning might capture these intricacies more effectively. A key methodological limitation stems from our use of LIWC-derived metrics rather than raw text data. While this structured approach allowed for analysis of linguistic dimensions through traditional machine learning methods, it also abstracts raw text into categorical scores, meaning our findings reflect preprocessed linguistic features rather than direct social media discourse.

Nevertheless, our results, which were derived from classifying sentiments constructed using LIWC, were still impressive (70%). Future investigations should explore the synergistic potential of combining LIWC and machine learning for classifying social media data. In addition, future work should consider advanced natural language processing techniques to better bridge the gap between computational classification and the complexities of natural language use in public health contexts. While we observed linguistic variations between HDS and LDS, the effect sizes were modest (Cohen d=0.04-0.19) [50]. Centering the study on states with the highest and lowest opioid-related death rates may inadvertently omit the multifaceted nature of chronic pain experiences throughout the United States. Aspects such as health care access, socioeconomic dynamics, and cultural nuances might influence chronic pain experiences and their linguistic expression across regions. By concentrating solely on these states, we might have bypassed crucial regional differences and insights.

Several future directions emerge from this work. Subsequent studies might consider using daily diary studies that incorporate similar linguistic markers. While past research has executed daily diary studies on pain, such as that by Burns et al [14], none have integrated daily diary methods with the use of LIWC and the recently identified pain dictionary from Wright et al [9]. In addition, to refine linguistic markers and categories, future qualitative research could be instrumental in delving deeper into the emotions and thoughts associated with pain. This is particularly relevant for diverse populations given the significant influence of culture on pain experiences [51]. Forthcoming research might explore advanced natural language processing techniques, such as recursive neural networks, to probe emotion-related phenomena tied to chronic pain. Such methodologies might offer benefits over word count-based strategies such as LIWC [52].

The prolific use of Twitter for disseminating pain experiences and coping methods has offered invaluable insights into the nuanced narratives of pain within a social context. The ability of linguistic features in pain-related tweets to predict opioid mortality, as delineated in this paper, emphasizes the potential value of the social communication model of pain. These findings align with the growing field of opioid use surveillance, which leverages social media data to track patterns of opioid misuse and monitor public sentiment toward pain management and treatment. Although this is a preliminary foray in this domain, our results suggest the considerable impact of merging social factors with pain dialogue, thereby stressing the imperative for comprehensive, multidisciplinary research. Such endeavors could potentially lay the groundwork for all-encompassing pain management strategies, considering both the physiological and social aspects of chronic pain.

### Conclusions

This study accentuates the complex relationship between linguistic expressions of pain on platforms such as Twitter and the wider sociopsychological challenges confronting patients with chronic pain. Our findings contribute to the broader discourse on narrative medicine, demonstrating that individuals not only share pain-related symptoms but also construct social narratives that reflect distress, coping strategies, and health care experiences. These insights further emphasize the role of opioid use surveillance in understanding digital pain discourse, as social media platforms provide a unique avenue for identifying opioid-related risks and public health trends. However, given the challenges of distinguishing user-generated content from external influences, such as media coverage or public health campaigns, future research should consider refining methodological approaches to better isolate individual experiences from broader societal narratives.

Understanding these narratives could inform clinician training, patient–health care provider communication, and the development of digital interventions that align with patient-centered care approaches. Future work should examine how patients actively engage in digital storytelling and whether such engagement can support narrative medicine practices in pain management and opioid use interventions. Grounding our perspective in the social communication model of pain, it became evident that conversations about chronic pain go beyond just physiological experiences, intertwining profoundly with social narratives and viewpoints. The linguistic predictors, particularly concerning opioid mortality rates across varied US states, spotlight the significant ramifications of these social narratives. Harnessing the power of machine learning and semantic network analysis, we not only unveil nuances in the discourse of pain but also stress the necessity of integrating social factors into strategies for pain management and addressing the opioid crisis.

## Data Availability

The data used in this study are publicly available from Twitter. Data can be collected from Twitter (now X) using their application programming interface as described in the Methods section. The datasets generated or analyzed during this study are available from the corresponding author on reasonable request.

## Abbreviations

API: application programming interface
CB: betweenness centrality
CC: closeness centrality
CD: degree centrality
CE: eigenvector centrality
DN: node degree
HDS: high-death states
LDS: low-death states
LIWC: Linguistic Inquiry and Word Count
SMOTE: Synthetic Minority Oversampling Technique
